# Cost-Effectiveness of Treating Hepatitis C with Sofosbuvir/Ledipasvir in Germany

**DOI:** 10.1371/journal.pone.0169401

**Published:** 2017-01-03

**Authors:** Jona T. Stahmeyer, Siegbert Rossol, Sebastian Liersch, Ines Guerra, Christian Krauth

**Affiliations:** 1 Institute for Epidemiology, Social Medicine and Health Systems Research; Hannover Medical School; Hannover; Germany; 2 Department of Internal Medicine; Krankenhaus Nordwest; Steinbacher Hohl 2–26; Frankfurt am Main; Germany; 3 Real World Strategy and Analytics; MAPI Group; 3rd Floor Beaufort House; Uxbridge, Middlesex; United Kingdom; Universita degli Studi di Pisa, ITALY

## Abstract

**Background:**

Infections with the hepatitis C virus (HCV) are a global public health problem. Long-term consequences are the development of liver cirrhosis and hepatocellular carcinoma. Newly introduced direct acting antivirals, especially interferon-free regimens, have improved rates of sustained viral response above 90% in most patient groups and allow treating patients who were ineligible for treatment in the past. These new regimens have replaced former treatment and are recommended by current guidelines. However, there is an ongoing discussion on high pharmaceutical prices. Our aim was to assess the long-term cost-effectiveness of treating hepatitis C genotype 1 patients with sofosbuvir/ledipasvir (SOF/LDV) treatment in Germany.

**Material and Methods:**

We used a Markov cohort model to simulate disease progression and assess cost-effectiveness. The model calculates lifetime costs and outcomes (quality-adjusted life years, QALYs) of SOF/LDV and other strategies. Patients were stratified by treatment status (treatment-naive and treatment-experienced) and absence/presence of cirrhosis. Different treatment strategies were compared to prior standard of care. Sensitivity analyses were performed to evaluate model robustness.

**Results:**

Base-case analyses results show that in treatment-naive non-cirrhotic patients treatment with SOF/LDV dominates the prior standard of care (is more effective and less costly). In cirrhotic patients an incremental cost-effectiveness ratio (ICER) of 3,383 €/QALY was estimated. In treatment-experienced patients ICERs were 26,426 €/QALY and 1,397 €/QALY for treatment-naive and treatment-experienced patients, respectively. Robustness of results was confirmed in sensitivity analyses.

**Conclusions:**

Our analysis shows that treatment with SOF/LDV is cost-effective compared to prior standard of care in all patient groups considering international costs per QALY thresholds.

## Background

Worldwide up to 185 million people have been infected with the hepatitis C virus (HCV) and as a result of the infection and long-term consequences 350,000 people die every year [1]. Estimates assume that about 27% of liver cirrhosis and 25% of hepatocellular carcinoma are attributable to chronic HCV [2]. Data from the German National Health and Examination Survey (DEGS1) show an anti HCV-prevalence of 0.3% in Germany [3]. Considering a higher prevalence in risk-groups such as drug abuser and prison inmates, recent studies estimate number of infected people at 275,000 [4]. The majority of the patients are infected with HCV genotype 1 or 3 [5]. A large part of infected patients are unaware of their disease and most infections remain undiagnosed until serious, and potentially lethal, complications such as liver cirrhosis and hepatocellular carcinoma occur [6].

The historical dual therapy with pegylated interferon and ribavirin (PegIFN/RBV) was the standard of care for a decade until first generation protease inhibitors telaprevir (TVR) and boceprevir (BOC) were approved for the treatment of patients with HCV genotype 1 in 2011. Treatment options significantly improved with the introduction of first interferon-free treatment regimens starting in SOF in January 2014. Other direct acting antivirals (DAAs) simeprevir (SMV), daclatasvir (DCV), sofosbuvir/ledipasvir (SOF/LDV) and combination treatment with ombitasvir/paritaprevir/ritonavir and dasabuvir (OMV/PTV/RTV+DSV) subsequently followed. New treatments have sustained virologic response (SVR) rates of >90%, a favorable toxicity profile, a shorter treatment duration and thus enable to cure most patients. New DAAs are recommended for treatment by current AASLD, EASL and German guidelines whereby the latter primarily focus on interferon-free treatments which have been evaluated in phase-3 trials. In genotype 1 patients only treatment with SOF/LDV±RBV and OMV/PTV/RTV+DSV±RBV are recommended without any restrictions. Therapeutic advances were accompanied by an increase in treatment costs raising the question if high costs are justified and affordable for healthcare systems, even in western countries. After introduction, several European countries have limited the access to new DAAs and primarily treat patients with advanced HCV-infection in order to control HCV-related health expenditure.

Up to now, several analyses have estimated cost-effectiveness of newly introduced treatment regimens [7–10]. Nevertheless, studies including recently introduced interferon-free regimens SOF/LDV±RBV and OMV/PTV/RTV+DSV±RBV are rare and usually refer to the US healthcare system. Furthermore, latest guideline recommendation on treatment regimen considering treatment duration and the use of RBV are usually not considered.

For example, Younossi et al. (2015) analyzed cost-effectiveness of SOF/LDV regimens in patients with genotype 1 infection [7]. They showed that LDV/SOF treatment dominates all previous treatments in treatment-naive and treatment-experienced patients, except for treatment with SOF/SMV which shows marginally better outcomes but considerably higher costs. Treatment with OMV/PTV/RTV+DSV was not considered [7]. Two other US studies evaluating SOF/LDV treatment determined higher lifetime costs and better outcomes compared to old standard of care. Both studies conclude that SOF/LDV could be considered as cost-effective [8;9].

The aim of the present study was to assess the long-term cost-effectiveness of treating chronic hepatitis C genotype 1 patients with SOF/LDV in Germany based on current German guidelines.

## Methods

### Study Design

We used a Markov cohort model to determine the cost-effectiveness (long-term outcomes and costs) of treating chronic hepatitis C genotype 1 patients with sofosbuvir/ledipasvir ± Ribavirin (LDV/SOF±RBV). The study was conducted from the perspective of the German healthcare system. An annual discount-rate of 3% was used as recommended by the German Institute for Quality and Efficiency in Health Care (IQWiG) [11]. The model was programmed with Microsoft Excel.

### Outcomes

To analyze long-term cost-effectiveness we calculated lifetime costs and quality-adjusted life years (QALYs) and derived incremental cost-effectiveness ratios (ICER) for different treatment strategies.

### Study Population

Our base-case was a hypothetical cohort of 10,000 patients. Treatment-naive patients initiate treatment at an age of 40 years and treatment-experienced patients at an age of 45 years. Patients were stratified according to the presence/absence of liver cirrhosis. We assumed an average weight of 80kg for weight-based medications.

### Treatment Strategies and Effectiveness

Treatment strategies of LDV/SOF±RBV as well as alternative regimens depend on treatment status (treatment-naive vs. treatment-experienced) and presence/absence of cirrhosis. Treatment-naive non-cirrhotic patients received 8 or 12 weeks of LDV/SOF depending on baseline viral load; cirrhotic patients received 12 weeks of LDV/SOF+RBV. We assumed, based on data from the German Hepatitis C Registry, that 90.7% of treatment-naive patients had viral load <6 Mio IU/ml and qualify for 8 weeks of treatment. Treatment experienced non-cirrhotic patients received 12 weeks of LDV/SOF and cirrhotic patients additionally received RBV. Alternative treatment strategies and effectiveness data are summarized in Table 1. Treatments are based on current German guidelines and EASL guidelines. Effectiveness data for each patient group was derived from clinical trials and summary of product characteristics. We assumed a GT-1a/b ratio of 47%/53% based on recent data [12].

**Table 1:** Treatment regimens.

Treatment regimen		Treatment duration	SVR-rate (Range)	Source:
	**Treatment naive**			
	**non-cirrhotic:**			
	SOF/LDV	8 or 12 weeks	96.8% (91.8% - 100.0%)	[13–15]
	SOF + PegIFN + RBV	12 weeks	92.3% (88.5% - 95.2%)	[16;17]
	PTV/r/OMV/DSV ± RBV	12 weeks	98.1% (93.1% - 99.9%)	[18–20]
	SOF+RBV	24 weeks	67.6% (60.0% - 75.0%)	[17]
	SMV + PegIFN + RBV	24 weeks	82.0% (78.0% - 85.0%)	[21–23]
	SOF + DCV	12 weeks	100.0% (95.0% - 100.0%)	[24;25]
	SOF + SMV	12 weeks	94.1% (79.4% - 99.8%)	[26]
	TVR + PegIFN + RBV	24 or 48 weeks	77.3% (74.8% - 79.7%)	[27]
	BOC + PegIFN + RBV	28 or 48 weeks	64.1% (60.2% - 67.9%)	[28]
	PegIFN + RBV	48 weeks	43.6% (40.3% - 46.9%)	[29]
	**cirrhotic:**			
	SOF/LDV +RBV	12 weeks	98.0% (94.5% - 99.8%)	[15;30]
	SOF + PegIFN + RBV	12 weeks	79.6% (66.5% - 89.4%)	[16;17]
	PTV/r/OMV/DSV + RBV	12 (1b) or 24 weeks (1a)	97.5% (88.9% - 99.1%)	[18–20]
	SOF+RBV	24 weeks	36.4% (12.2% - 65.2%)	[17]
	SMV + PegIFN + RBV	24 weeks	60.4% (46.4% - 73.6%)	[21–23]
	SOF + DCV	12 weeks	100.0% (95.0% - 100.0%)	[24;25]
	SOF+SMV	12 weeks	92.9% (66.5% - 99.9%)	[26]
	TVR + PegIFN + RBV	24 or 48 weeks	53.4% (44.9% - 61.9%)	[27]
	BOC + PegIFN + RBV	48 weeks	55.0% (42.4% - 67.3%)	[28]
	PegIFN + RBV	48 weeks	23.6% (16.2% - 32.0%)	[29]
	**Treatment experienced**			
	**non-cirrhotic:**			
	SOF/LDV	12 weeks	95.4% (90.1% - 98.7%)	[15;31]
	SOF + PegIFN + RBV	12 weeks	78.0% (68.0% - 88.0%)	[32]
	PTV/r/OMV/DSV ± RBV	12 weeks	98.1% (91.3% - 99.0%)	[33;34]
	SMV + PegIFN + RBV	48 weeks	76.5% (71.6–81.0%)	[23;35;36]
	SOF + DCV	12 weeks	95.4% (90.1% - 98.7%)	Assumption: equal to SOF/LDV
	SOF + SMV	12 weeks	92.9% (66.5% - 99.9%)	[23;26]
	TVR + PegIFN + RBV	24 or 48 weeks	72.2% (65.7% - 78.2%)	[37;38]
	BOC + PegIFN + RBV	48 weeks	64.4% 56.1% - 72.3%)	[39;40]
	PegIFN + RBV	48 weeks	17.6% (10.9% - 25.6%)	[37;38]
	**cirrhotic:**			
	SOF/LDV +RBV	12 weeks	96.1% (90.8% - 99.2%)	[15;30]
	SOF + PegIFN + RBV	12 weeks	71.0% (61.0% - 81.0%)	[32]
	PTV/r/OMV/DSV + RBV	12 (1b) or 24 weeks (1a)	96.7% (90.8% - 98.9%)	[20;41]
	SMV + PegIFN + RBV	48 weeks	66.7% (54.7% - 77.7%)	[23;35;36]
	SOF + DCV	12 weeks	86.4% (69.6% - 97.0%)	Assumption: equal to SOF/LDV
	SOF+SMV	12 weeks	92.9% (66.5% - 99.9%)	[23;26]
	TVR + PegIFN + RBV	48 weeks	47.2% (35.9% - 58.7%)	[37;38]
	BOC + PegIFN + RBV	48 weeks	35.3% (15.2% - 58.7%)	[39;40]
	PegIFN + RBV	48 weeks	10.0% (2.2% - 22.8%)	[37;38]

### Model Structure

**Fig 1:**
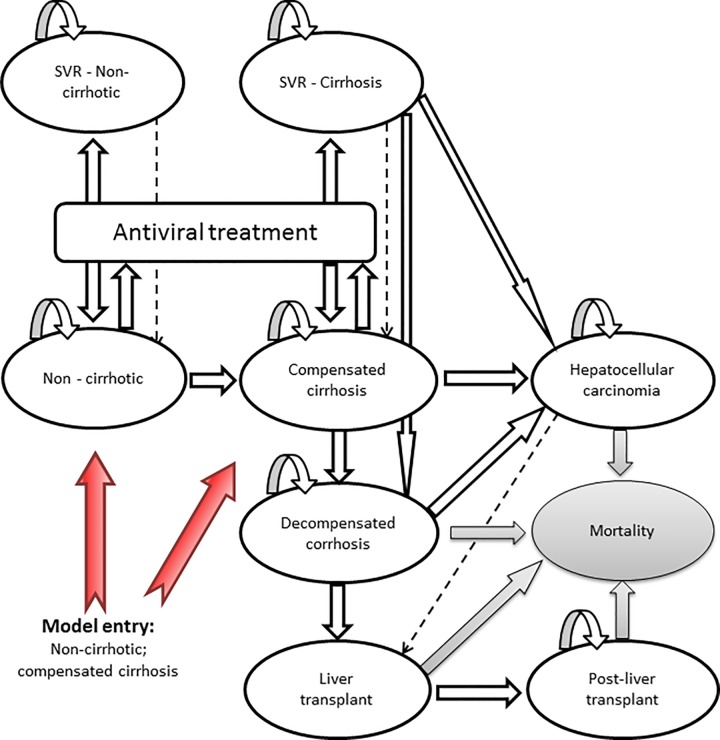
Model schematic.

The model reflects the natural course of the infection and simulates the lifetime progression of patients with chronic HCV infection. After initiating treatment patients were followed over a lifetime (lifetime horizon). Progression is characterized by different stages of disease severity based on the METAVIR score (Fig 1).

In non-cirrhotic patients disease progression is stopped after achieving SVR. Patients with cirrhosis could develop decompensated cirrhosis or hepatocellular carcinoma even after achieving SVR whereas probability of progression is significantly reduced compared to patients without SVR. To facilitate the modeling of different treatment strategies and the transition to SVR, cycle-length is monthly for the first one and a half years and next two cycles are quarterly. From year two after initiation of treatment, transition occurs on a yearly basis. We assumed a split for mild and moderate patients based on the proportion of patients with a METAVIR score of F0-F2 (mild) and F3 (moderate) in ION trials, which is 78% and 22%.Transition probabilities were derived from published literature Table 2 [42–46]. All-cause mortality rates were derived from the German Federal Statistical Office [47].

**Table 2:** Model inputs.

Transition probabilities		Base-Case	Range/Variation	Source
**Health State**				
**From:**	**To:**	**Annual transition probability**		
Non-cirrhotic	Compensated cirrhosis (age 30 years)	0.006	0.003–0.010	[42]
	Compensated cirrhosis (age 40 years)	0.009	0.005–0.015	[42]
	Compensated cirrhosis (age 50 years)	0.016	0.008–0.026	[42]
Compensated cirrhosis	Decompensated cirrhosis	0.044	0.029–0.058	[43]
	Hepatocellular carcinoma	0.063	0.046–0.080	[43]
Compensated cirrhosis (SVR)	Decompensated cirrhosis	0.006	0.000–0.013	[43]
	Hepatocellular carcinoma	0.013	0.003–0.022	[43]
Decompensated cirrhosis	Hepatocellular carcinoma	0.014	0.002–0.039	[44]
	Liver Transplant	0.022	0.011–0.033	[45]
	Death	0.130	0.111–0.150	[44]
Hepatocellular carcinoma	Death	0.430	0.372–0.489	[44]
Liver transplant	Death	0.210	0.127–0.307	[46]
Post Liver Transplant	Death	0.057	0.037–0.082	[46]
**Quality of life utilities**				
**Health State**		**Utility**		
Non Cirrhotic		0.81	0.73–0.90	[48]
Compensated Cirrhosis		0.74	0.67–0.78	[48]
Decompensated Cirrhosis		0.72	0.66–0.79	[48]
Hepatocellular Carcinoma		0.72	0.66–0.79	[48]
Liver Transplant		0.72	0.66–0.79	[48]
Post Liver Transplant		0.79	0.66–0.92	[48]
QoL increase if SVR is achieved		0.041	0.024–0.063	[49]
QoL Increment SOF/LDV		+ 4.43%	+3.30% to +5.73%	Data on file
QoL Increment SOF/LDV + RBV		- 3.25%	-1.55% to -5.56%	Data on file
QoL Increment SOF + PegIFN + RBV		- 14.52%	-11.15% to -18.33%	Data on file
QoL Increment TVR + PegIFN + RBV		- 14.27% (TN) / - 14.61% (TE)	-12.37% to -16.29%	[50]
QoL Increment BOC + PegIFN + RBV		- 12.20%	-10.28% to 14.28%	[51]
QoL Increment PegIFN + RBV		- 14.75%	-6.31% to -26.71%	[46]
SOF + DAC		+ 4.43%	+3.30% to +5.73%	Assumption: equal to SOF/LDV
SOD + SMV		+ 4.43%	+3.30% to +5.73%	Assumption: equal to SOF/LDV
QoL Increment SMV + PegIFN + RBV		- 14.27% (TN) / - 14.61% (TE)	-12.37% to -16.29%	Assumption: equal to TVR + PegIFN + RBV
QoL Increment PTV/r/OMV/DSV		+ 4.43%	+3.30% to +5.73%	Assumption: equal to SOF/LDV
QoL Increment PTV/r/OMV/DSV + RBV		- 3.25%	-1.55% to -5.56%	Assumption: equal to SOF/LDV + RBV
**Pharmaceuticals, € per week**				[52]
SOF/LDV		4,721	no variation	
SOF		3,963	no variation	
DAC		2,241	no variation	
SMV		2,339	no variation	
BOC		786	no variation	
TVR		2,339	no variation	
PegIFN-α2a		254	no variation	
PegIFN-α2b		274	no variation	
RBV		177	no variation	
OMV/r/PTV		3,807	no variation	
DSV		372	no variation	
**Health state costs, € per year**				[53;54]
Mild HCV		153	± 25%	
Moderate HCV		157	± 25%	
Compensated cirrhosis		776	± 25%	
Decompensated cirrhosis		9,768	± 25%	
HCC		24,096	± 25%	
Liver transplantation incl. 1^st^ year		143,480	± 25%	
Liver transplantation follow-up >1^st^ year		20,751	± 25%	
**On treatment costs**				Updated from [55]
Baseline diagnostics non-cirrhotic		298	no variation	
Baseline diagnostics cirrhotic		305	no variation	
Monitoring 8 weeks non PR		284	no variation	
Monitoring 12 weeks non PR		412	no variation	
Monitoring 24 weeks non PR		568	no variation	
Monitoring 12 weeks with PegIFN and/or RBV		431	no variation	
Monitoring 24 weeks with PegIFN and/or RBV		592	no variation	
Monitoring 48 weeks with PegIFN and/or RBV		800	no variation	
**Adverse event management costs, €**				
Nausea		31	no variation	
Vomiting		31	no variation	
Diarrhoea		48	no variation	
Pruritus		15	no variation	
Rash		33	no variation	
Anaemia (blood transfusion)		19	no variation	
Depression		7	no variation	

### Quality of Life Data

Health state utilities reflect the quality of life of HCV-infected patients in each health state on a scale from 1 (perfect health) to 0 (death). Information was derived from a study by Siebert et al (2003) who used EuroQoL-5D for determination of quality of life in HCV patients [48]. Information on reduction in quality of life during antiviral treatment was derived from clinical study reports or published literature. If no information were available for certain therapies, data from other treatments were used as an approximation (Table 2).

### Cost Data

Costs of treating hepatitis C included treatment associated costs and costs of disease progression (health state costs). Treatment costs comprise pharmaceuticals, costs for diagnostic procedures related to basic diagnostics for treatment initiation and on-treatment monitoring as well as adverse event management. Information on pharmaceutical costs were derived from the German drug directory (Lauer-Taxe) [52]. Costs for diagnostic procedures were adapted and updated from a published study on guideline-based treatment costs [55]. Costs for adverse event management covers prescribed medication on expert opinion. Health state costs were derived from published literature and inflated to 2015 [53;54;56]. Cost data are summarized in Table 2.

### Sensitivity Analyses

We performed deterministic (DSA) and probabilistic sensitivity analyses (PSA) to evaluate the robustness of the model and to examine the effect of uncertainty on primary outcomes. For variation of SVR-rates, quality of life and transition probabilities 95%-confidence intervals were used or calculated based on distribution assumptions. Health state costs were varied by ±25% as no detailed information on point estimates is available. Single components of treatment costs (basic diagnostic procedures, monitoring, pharmaceuticals and adverse event management costs) were not varied separately but varied as a whole by ±25%. The PSA is based on Monte Carlo simulation with 1,000 iterations. In this analysis all variables are varied simultaneously according to their distributions. We assumed beta distribution for SVR-rates, transition probabilities and quality of life utilities estimates. Gamma distribution was used for treatment costs, health state costs and utility decrements.

## Results

### Base-Case Results

The results of our base-case analysis were stratified for treatment-naive and treatment-experienced patients as well as absence/presence of liver cirrhosis and show average lifetime costs and outcomes for different treatment strategies (Table 3). Incremental cost effectiveness ratios were calculated comparing different regimens to recent standard of care with TVR +PR. Additionally, we compared SOF/LDV±RBV to other treatment regimens.

**Table 3:** Base-Case Analysis Results.

Treatment regimen	lifetime costs, €	QALYs	ICER (vs. TVR+PR), € per QALY	ICER (vs. SOF/LDV), € per QALY
**Treatment naive**				** **
**combined cohorts**				** **
SOF/LDV ± RBV	53.828	18,659	850	**reference**
SOF + PegIFN + RBV	62.912	18,271	13.692	~ dominated
PTV/r/OMV/DSV ± RBV	66.869	18,680	12.258	621.204
SOF+RBV	114.151	17,092	~ dominated	~ dominated
SMV + PegIFN + RBV	50.903	17,734	# dominant	3.162
SOF + DCV	83.608	18,750	25.351	324.749
SOF + SMV	85.664	18,530	33.047	~ dominated
TVR + PegIFN + RBV	52.875	17,538	**reference**	850
BOC + PegIFN + RBV	51.787	17,362	6.182	1.574
PegIFN + RBV	34.385	16,486	17.583	8.951
no treatment	22.138	15,358	14.101	9.602
**non-cirrhotic:**				
SOF/LDV	41.056	20,031	# dominant	**reference**
SOF + PegIFN + RBV	53.999	19,891	29.151	~ dominated
PTV/r/OMV/DSV ± RBV	52.490	20,061	17.280	381.843
SOF + RBV	103.696	19,307	~ dominated	~ dominated
SMV + PegIFN + RBV	41.322	19,629	# dominant	-661
SOF + DCV	75.557	20,104	55.290	475.904
SOF + SMV	77.398	19,971	75.541	~ dominated
TVR + PegIFN + RBV	43.073	19,516	**reference**	~ dominated
BOC + PegIFN + RBV	40.853	19,220	7.482	250
PegIFN + RBV	23.981	18,748	24.867	13.311
no treatment	11.559	17,831	18.704	13.408
**cirrhotic:**			** **	
SOF/LDV +RBV	93.185	14,429	3.383	**reference**
SOF + PegIFN + RBV	90.376	13,279	3.972	2.443
PTV/r/OMV/DSV + RBV	111.178	14,422	9.428	~ dominated
SOF + RBV	146.371	10,268	~ dominated	~ dominated
SMV + PegIFN + RBV	80.425	11,894	# dominant	5.034
SOF + DCV	108.418	14,580	8.075	100.955
SOF+SMV	111.136	14,092	10.589	~ dominated
TVR + PegIFN + RBV	83.080	11,442	**reference**	3.383
BOC + PegIFN + RBV	85.478	11,638	12.256	2.761
PegIFN + RBV	66.446	9,516	8.635	5.442
no treatment	54.737	7,738	7.651	5.746
**Treatment experienced**	** **	** **	** **	
**combined cohorts**				
SOF/LDV ± RBV	66.141	17,442	10.591	**reference**
SOF + PegIFN + RBV	62.836	16,764	19.149	4.879
PTV/r/OMV/DSV ± RBV	66.137	17,486	10.186	# dominant
SMV + PegIFN + RBV	53.655	16,654	# dominant	15.849
SOF + DCV	83.933	17,296	30.255	~ dominated
SOF + SMV	84.961	17,358	29.447	~ dominated
TVR + PegIFN + RBV	54.180	16,312	**reference**	10.591
BOC + PegIFN + RBV	54.757	16,037	~ dominated	8.102
PegIFN + RBV	30.325	14,961	17.658	14.440
no treatment	21.024	14,548	18.793	15.592
**non-cirrhotic:**			** **	
SOF/LDV	57.937	18,676	26.426	**reference**
SOF + PegIFN + RBV	53.956	18,296	62.251	10.471
PTV/r/OMV/DSV ± RBV	52.172	18,714	14.911	# dominant
SMV + PegIFN + RBV	44.433	18,192	15.552	27.893
SOF + DCV	75.723	18,676	58.796	~ dominated
SOF + SMV	77.165	18,626	67.563	~ dominated
TVR + PegIFN + RBV	43.417	18,127	**reference**	26.426
BOC + PegIFN + RBV	43.754	17,991	~ dominated	20.687
PegIFN + RBV	19.314	17,098	23.436	24.478
no treatment	10.515	16,795	24.701	25.205
**cirrhotic:**				
SOF/LDV +RBV	91.423	13,637	1.397	**reference**
SOF + PegIFN + RBV	90.201	12,044	2.155	767
PTV/r/OMV/DSV + RBV	109.174	13,703	7.319	270.711
SMV + PegIFN + RBV	82.075	11,914	# dominant	5.424
SOF + DCV	109.231	13,041	9.430	~ dominated
SOF+SMV	108.985	13,448	7.934	~ dominated
TVR + PegIFN + RBV	87.349	10,721	**reference**	1.397
BOC + PegIFN + RBV	88.664	10,015	~ dominated	762
PegIFN + RBV	64.254	8,376	9.849	5.164
no treatment	53.410	7,624	10.961	6.322

~ dominated: treatment is more expensive and less effective

# dominant: treatment is less expensive and more effective

In treatment-naive patients SOF/LDV±RBV (18.659 QALYs), PTV/r/OMV+DSV±RBV (18.680 QALYs) and SOF+DCV (18.750 QALYs) had highest long-term outcomes. Total lifetime costs were €53,828 (SOF/LDV±RBV), €66,869 (PTV/r/OMV+DSV±RBV) and €83,608 (SOF+DCV). Treating non-cirrhotic patients resulted in higher outcomes and lower total costs compared to cirrhotic patients. ICER compared to previous standard of care (TVR+PegIFN+RBV) was 850 €/QALY for SOF/LDV±RBV, 12,258 €/QALY for PTV/r/OMV+DSV±RBV and 25,351 €/QALY for SOF+DCV. Only treatment with SMV+PegIFN+RBV dominated TVR+PegIFN+RBV (showed higher outcomes and lower costs).

In treatment-experienced patients, regimens containing SOF/LDV±RBV (17.442 QALYs), PTV/r/OMV+DSV±RBV (17.486 QALYs) and SOF+SMV (17.358 QALYs) showed highest outcomes. Average lifetime costs of €66,141 (SOF/LDV±RBV), €66,137 (PTV/r/OMV+DSV±RBV) and €84,961 (SOF+SMV) were calculated. Just as in treatment-naive patients, outcomes were higher and lifetime costs lower in non-cirrhotic treatment-experienced patients. We calculated ICERs of 11.961 €/QALY for SOF/LDV±RBV, 11,957 €/QALY for PTV/r/OMV+DSV±RBV and 30,781 €/QALY for SOF+SMV compared to previous standard of care (TVR+PegIFN+RBV). Stratified data for non-cirrhotic and cirrhotic patients are shown in Table 3.

Compared to prior standard of care, higher SVR-rates in SOF/LDV±RBV and other DAA regimens can help to avoid the development of liver cirrhosis and end-stage liver disease in many patients (Table 4). In non-cirrhotic treatment-naive patients treatment with SOF/LDV can prevent the development of 58 cases of liver cirrhosis, 19 cases of decompensated liver cirrhosis, 28 cases of HCC and 2 liver transplants per 1,000 patients treated compared to prior standard of care (TVR+PegIFN+RBV). Data for cirrhotic patients show that SOF/LDV+RBV (vs. SoC) can prevent 97 cases of decompensated liver cirrhosis, 104 cases of HCC and 13 liver transplants per 1,000 patients treated.

**Table 4:** Prevented cases of liver cirrhosis, decompensated cirrhosis, HCC and liver transplant compared to SoC (per 1,000 patients treated).

Patient group	Cirrhosis	Decompensated Cirrhosis	HCC	Liver transplant
**Treatment naive patients**				
**non-cirrhotic:**				
SOF/LDV	-58	-19	-28	-2
SOF + PegIFN + RBV	-44	-14	-22	-2
PTV/r/OMV/DSV ± RBV	-62	-20	-30	-2
SOF + RBV	29	10	14	1
SMV + PegIFN + RBV	-14	-4	-7	0
SOF + DCV	-68	-22	-33	-2
SOF + SMV	-50	-16	-25	-2
TVR + PegIFN + RBV	**ref**	**ref**	**ref**	**ref**
BOC + PegIFN + RBV	39	13	19	1
PegIFN + RBV	99	32	49	4
no treatment	232	76	114	8
**cirrhotic:**				
SOF/LDV + RBV	n.a.	-97	-104	-13
SOF + PegIFN + RBV	n.a.	-57	-61	-8
PTV/r/OMV/DSV ± RBV	n.a.	-96	-104	-13
SOF + RBV	n.a.	38	41	5
SMV + PegIFN + RBV	n.a.	-15	-16	-2
SOF + DCV	n.a.	-102	-109	-14
SOF + SMV	n.a.	-86	-92	-11
TVR + PegIFN + RBV	n.a.	**ref**	**ref**	**ref**
BOC + PegIFN + RBV	n.a.	-4	-5	-1
PegIFN + RBV	n.a.	65	70	9
no treatment	n.a.	118	128	16
**Treatment experienced patients**				
**non-cirrhotic:**				
SOF/LDV	-62	-19	-29	-2
SOF + PegIFN + RBV	-15	-5	-7	0
PTV/r/OMV/DSV ± RBV	-69	-22	-33	-2
SMV + PegIFN + RBV	-12	-4	-6	0
SOF + DCV	-62	-19	-29	-2
SOF + SMV	-55	-17	-26	-2
TVR + PegIFN + RBV	**ref**	**ref**	**ref**	**ref**
BOC + PegIFN + RBV	20	6	10	1
PegIFN + RBV	146	46	69	5
no treatment	195	62	93	7
**cirrhotic:**				
SOF/LDV + RBV	n.a.	-110	-123	-14
SOF + PegIFN + RBV	n.a.	-53	-58	-7
PTV/r/OMV/DSV + RBV	n.a.	-111	-125	-15
SMV + PegIFN + RBV	n.a.	-45	-51	-6
SOF + DCV	n.a.	-88	-98	-11
SOF + SMV	n.a.	-102	-114	-13
TVR + PegIFN + RBV	n.a.	**ref**	**ref**	**ref**
BOC + PegIFN + RBV	n.a.	27	31	4
PegIFN + RBV	n.a.	85	97	11
no treatment	n.a.	109	125	14

n.a.–not applicable; ref–reference treatment

In non-cirrhotic treatment-experienced patients treatment with SOF/LDV can prevent the development of 62 cases of liver cirrhosis, 19 cases of decompensated liver cirrhosis, 29 cases of HCC and 2 liver transplants per 1,000 patients treated compared to TVR+PegIFN+RBV. Data for cirrhotic patients show that SOF/LDV+RBV can prevent 110 cases of decompensated liver cirrhosis, 123 cases of HCC and 14 liver transplants per 1,000 patients treated.

### Sensitivity Analysis

**Fig 2:**
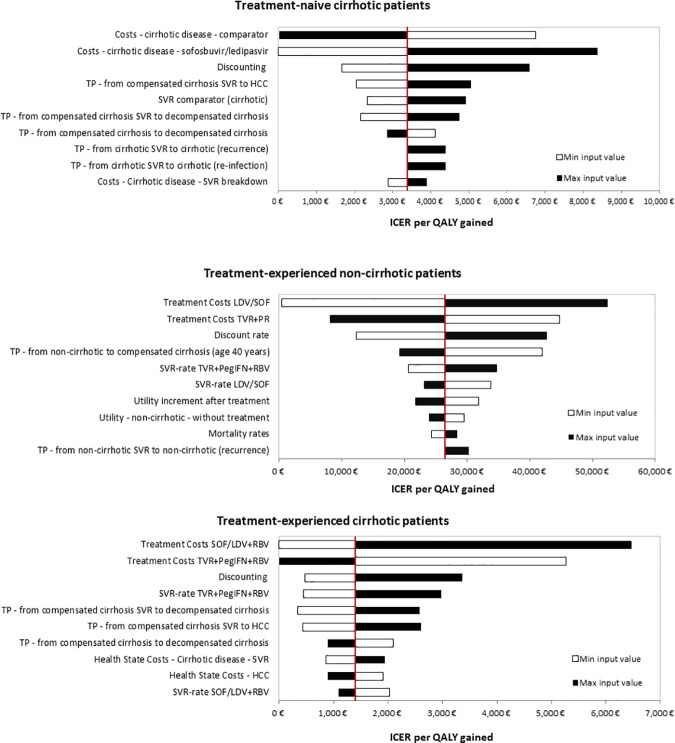
Deterministic Sensitivity Analysis Results.

In one-way sensitivity analysis, we identified the ten variables which have the largest impact on costs per QALY results (SOF/LDV±RBV vs. TVR+PegIFN+RBV). Analyses were performed separately for different subgroups. In treatment-naive non-cirrhotic patients only variation on treatment costs for SOF/LDV and treatment comparator had significant impact leading to the fact of SOF/LDV did not dominate the comparator anymore. In treatment-naive cirrhotic patients, treatment costs of SOF/LDV+RBV and TVR+PegIFN+RBV as well as the discount rate had highest impact on study results. In treatment-experienced patients, highest impacts on results were observed for treatment costs and discount rate as well (Fig 2).

Robustness of base-case results was confirmed in PSA. Results for treatment-naive non- cirrhotic patients showed that the chance of SOF/LDV for being more effective and less costly is 100%. In treatment-naive patients with cirrhosis treatment with SOF/LDV+RBV induced in higher costs and higher outcomes in any case (vs. TVR+PegIFN+RBV). The probability of being cost-effective at a willingness-to-pay threshold of €30,000 is 100%. In treatment-experienced non-cirrhotic patients the probability of SOF/LDV being cost-effective was 60% at a threshold of 30,000 €/QALY and 92% at 40,000 €/QALY (vs. TVR+PegIFN+RBV); in cirrhotic patients the probability of SOF/LDV+RBV being cost-effective was 100% at a threshold of 30,000 €/QALY.

## Discussion

The introduction of direct acting antivirals was the milestone in the treatment of chronic hepatitis C. Newly introduced treatment regimens have substantially increased SVR-rates, shortened treatment duration and show a favorable toxicity profile. Furthermore, these regimens allow curing patients who could not be treated previously (e.g. due to interferon intolerance, advanced cirrhosis, comorbidities).

We analyzed cost-effectiveness of SOF/LDV±RBV for the treatment of HCV genotype 1 patients. Analyses were conducted for treatment-naive and treatment-experienced patients considering presence/absence of liver cirrhosis. This regimen is recommended by the current German guideline as well as the regimen containing PTV/r/OMV/DSV±RBV and was also included our analyses [57].

Results show that treatment with SOF/LDV±RBV is cost-effective compared to prior standard of care considering a willingness-to-pay threshold of €30,000. The regimen of SOF/LDV even is cost saving (showed better outcomes and lower costs) in treatment-naive non-cirrhotic patients as a large part only require treatment for 8 weeks; treatment in cirrhotic patients resulted in 3,383 €/QALY. Incremental cost-effectiveness ratios were 26,426 €/QALY in non-cirrhotic and 1,397 €/QALY in cirrhotic treatment-experienced patients. Results were robust in multiple sensitivity analyses.

Several international studies have analyzed cost-effectiveness of newly introduced direct acting antivirals, especially for the US, but have not been performed for the German setting yet.

Najafzadeh et al (2015) analyzed cost-effectiveness of novel treatment regimens in treatment-naive hepatitis C patients. In genotype 1 patients, different strategies were compared to the previous standard of care (BOC+PegIFN+RBV). Treatment with SOF/LDV for 12 weeks resulted in an incremental cost-effectiveness ratio of 12,825 $/QALY. Other interferon-free regimens showed less favorable results (12 weeks SMV/SOF: 71,445 $/QALY; 12 weeks DCV/SOF: 63,355 $/QALY). The authors conclude that new treatment regimens represent good long-term economic value in genotype 1 patients [9].

Results from another US study by Chhatwal et al. (2015) evaluated cost-effectiveness of SOF/LDV (8 or 12 weeks) compared to the old standard of care (TVR+ PegIFN+RBV and BOC+PegIFN+RBV). The analyses showed incremental cost-effectiveness ratios of 31,452 $/QALY for non-cirrhotic and 9,703 $/QALY for cirrhotic treatment-naive patients. In treatment-experienced patients 35,853 $/QALY and 79,238 $/QALY were estimated for non-cirrhotic and cirrhotic patients, respectively. Assuming a willingness-to-pay threshold of 50,000 €/QALY treatment of genotype 1 patients with SOF/LDV is cost-effective in most patient groups [8].

A study by Younossi et al. (2015) comes to the conclusion that SOF/ treatment LDV (8,12 or 24 weeks depending on patient characteristics) dominates other treatment strategies except for SOF+SMV (12 or 24 weeks), which provides slightly better results but is considerably more expensive. Sensitivity analyses show that costs of alternative treatment strategies have the greatest impact on study results [7].

Zhang et al. (2015) analyzed cost-effectiveness of recently introduced regimens, primarily considering treatment-naive patients [10]. In non-cirrhotic genotype 1 patients both, SOF/LDV (12 weeks) and OMV/PTV/RTV + DSV + RBV (12 weeks), dominate triple-therapy with TVR + PegIFN/RBV the prior standard of care (higher effectiveness and lower lifetime costs). In patients with cirrhosis SOF/LDV (12 weeks) dominates TVR+PegIFN+RBV, whereas treatment with OMV/PTV/RTV+DSV+RBV (12 weeks) shows an incremental cost-effectiveness ratio of 25,227 $/QALY [10].

In comparing different studies, it has to be taken into account that transferability of economic evaluations is limited since healthcare systems, structures of care provision and remuneration schemes differ considerably between countries [58]. The comparability is made even more difficult considering differences in study design like modeling approaches, patient characteristics and treatment strategies. Therefore, there is a high need for the defining of up-to-date national data. Nevertheless, there are comparable results such as the dominant factor of treatment costs on study results. Most studies prove that treatment with SOF/LDV is cost-effective.

There are some limitations that have to be taken into account when interpreting the results of our study. Efficacy data is based on the results from different clinical trials and data for certain treatment regimens are based on a relatively small patient samples. Usually SVR-rates from clinical trials are not easily transferable into clinical practice [59]. Nevertheless, recent data from real-world SVR-rates show comparable SVR-rates in clinical practice. In the TRIO study SVR-rates in treatment-naive non-cirrhotic patients of 95% for an 8-week treatment with SOF/LDV and 96% for a 12-week treatment with SOF/LDV±RBV were observed [60]. Data on treatment-experienced patients show SVR-rates of 84% for 12 weeks of SOF/LDV, 96% for 12 weeks of SOF/LDV+RBV and 92% for 24 weeks of SOF/LDV [61]. German data confirm high SVR-rates for newly introduced interferon-free regimens [62]. A general problem is the reliability and availability of data. Extensive literature analyses were performed to determine the best available data. Furthermore, the impact of special patient types (e.g. with certain comorbidities, alcohol or drug abuse) was insufficiently taken into account as data refers to average patients. To determine cost-effectiveness in special HCV populations separate analyses and modeling approaches are necessary.

Although several studies have shown that HCV treatment with new DAA is cost-effective or even cost-saving in certain patient groups, affordability for healthcare systems and payers is doubtful. In addition to high costs of newly introduced agents, new treatment options allow to treat and cure patients who were not eligible for treatment in the past (e.g. patients with certain comorbidities or advanced liver disease, interferon-intolerant). Therefore, the number of patients available for treatment increased significantly, which stresses healthcare budgets further. Even western countries have limited or delayed access to new treatment options and restricted the use for patients with advanced liver disease [63;64]. Prioritization of severely ill might be a short-term solution for reducing expenditure. However, it should be taken into account that a major goal of treating hepatitis C is to prevent the development of liver cirrhosis and its complications. Even if patients with liver cirrhosis are successfully treated, they are still at risk of decompensation or developing HCC [43].

Treatment with SOF/LDV is recommended by national and international guidelines. Our analyses showed that this treatment is cost-effective compared to the prior standard of care in genotype 1 patients (triple-therapy with TVR). Besides individual treatment costs, impact of new treatments on healthcare budgets should not be forgotten.

## Abbreviations

OMV: Ombitasvir
PTV: Paritaprevir
RTV: Ritonavir
DSV: Dasabuvir
RBV: Ribavirin
PegIFN: Peginterferon
SOF: Sofosbuvir
LDV: Ledipasvir
SMV: Simeprevir
DCV: Daclatasvir
TVR: Telaprevir
BOC: Boceprevir
QALY: Quality-adjusted life year
ICER: Incremental cost-effectiveness ratio
HCV: Hepatitis C virus
SVR: sustained virologic response
PSA: probabilistic sensitivity analyses
DSA: deterministic sensitivity analyses
SoC: Standard of care
